# Amount of physical activity necessary for a normal level of high-sensitivity C-reactive protein in ELSA-Brasil: a cross-sectional study

**DOI:** 10.1590/1516-3180.2019.0301.R2.20102019

**Published:** 2020-04-22

**Authors:** Ciro Oliveira Queiroz, Francisco Pitanga, Paulo Andrade Lotufo, Maria Del Carmen Bisi Molina, Estela Maria Leão de Aquino, Maria Conceição Chagas Almeida

**Affiliations:** I MSc. Assistant Professor, Department of Physical Education, Escola Bahiana de Medicina e Saúde Pública (EBMSP), Salvador (BA), Brazil. Postgraduate Student, Postgraduate Program in Biotechnology in Health and Investigative Medicine, Centro de Pesquisa Gonçalo Muniz (CPqGM), Fundação Oswaldo Cruz (FIOCRUZ), Salvador (BA), Brazil.; II PhD. Associate Professor, Department of Physical Education, Universidade Federal da Bahia (UFBA), Salvador (BA), Brazil.; III MD, PhD. Full Professor, Clinical and Epidemiological Research Center, Universidade de São Paulo (USP), São Paulo (SP), Brazil.; IV PhD. Associate Professor, Public Health Program, Universidade Federal do Espirito Santo (UFES), Vitória (ES), Brazil.; V MD, PhD. Full Professor, Institute of Collective Health, Universidade Federal da Bahia (UFBA), Salvador (BA), Brazil.; VI PhD. Public Health Researcher, Centro de Pesquisas Gonçalo Moniz (CPqGM), Fundação Oswaldo Cruz (FIOCRUZ), Salvador (BA), Brazil.

**Keywords:** Motor activity, Risk factors, ROC curve, Cardiovascular diseases, Inflammation, Exercises, Chronic diseases, ELSA-Brasil

## Abstract

**BACKGROUND:**

Studies have shown that physical activity levels can be inversely associated with high-sensitivity C-reactive protein (hs-CRP) levels. However, the amount of physical activity required to maintain normal hs-CRP levels is still a matter for speculation.

**OBJECTIVE:**

To identify the amount of physical activity necessary to discriminate the hs-CRP levels in adults.

**DESIGN AND SETTING:**

Cross-sectional study at six teaching and research institutions.

**METHODS:**

The study sample comprised 10,231 adults aged 35 to 74 years who were participants in the Brazilian Longitudinal Study of Adult Health (ELSA-Brasil). Receiver operating characteristic (ROC) curves were constructed to compare the amount of physical activity in two domains (leisure time and commuting) with hs-CRP levels. The sensitivity and specificity were calculated to identify the best cutoff for physical activity level that would be needed to maintain normal levels of hs-CRP (< 3 mg/l).

**RESULTS:**

The area under the ROC curve was only statistically significant for discriminating normal levels of hs-CRP according to the amount of physical activity when the two study domains were added together. The accumulated physical activity level of 200 minutes/week was the best cutoff for discriminating normal levels of hs-CRP in adults of both sex.

**CONCLUSIONS:**

Physical activity in the leisure-time and commuting domains together, of duration 200 minutes/week, was associated with normal hs-CRP values.

## INTRODUCTION

Cardiovascular disease is the leading cause of death and disability worldwide and in Brazil.^1^^,^^2^ Epidemiological studies conducted over the last 50 years have described risk factors for cardiovascular disease and for coronary heart disease, such as dyslipidemia, hypertension, smoking and diabetes.^3^ Moreover, new risk markers such as high-sensitivity C-reactive protein (hs-CRP) levels have been highlighted over recent years as potential predictors of cardiovascular risk.^4^^,^^5^

hs-CRP is an acute-phase protein produced in the liver through the primary stimulus of the interleukins IL-1 and IL-6. It has also been assumed that hs-CRP can be produced from the arterial wall. hs-CRP is an extremely sensitive marker for inflammation and tissue damage.^6^ Recently, chronic inflammation has been identified as a component in the development and progression of atherosclerosis.^7^^,^^8^^,^^9^

The association between regular physical activity and cardiovascular risk factors,^10^^,^^11^ including hs-CRP,^12^^,^^13^ has been the subject of evaluations. The MONICA (Multinational Monitoring of trends and determinants in Cardiovascular disease) study in Augsburg, Germany, among men and women aged 35 to 74 years, investigated the association between different domains of physical activity (leisure-time, work, domestic and commuting domains) and markers for inflammation (fibrinogen, hs-CRP and IL-6). An inverse association was found between total physical activity and hs-CRP, even after adjusting for potential confounders.^14^^,^^15^ Other studies have shown similar results, i.e. indicating significant reduction of hs-CRP levels when moderate exercise is performed.^16^^,^^17^

Physical activity is defined as any bodily movement produced by skeletal muscles that results in energy expenditure above resting levels. It may occur within the domains of leisure time, commuting, household or occupational activities.^18^ The amount of physical activity that is required to maintain hs-CRP at normal levels has not yet been established and few studies have provided such information for populations in low and middle-income countries, including Brazil, where chronic non-transmitted diseases comprise the greatest burden on morbidity and mortality rates.^1^

The Brazilian Longitudinal Study of Adult Health (ELSA-Brasil) is a multicenter cohort conducted on adults in Brazil aged 35 to 74. The aim of ELSA-Brasil is to evaluate the complex web of risk factors associated with cardiovascular disease and diabetes, which includes physical activity and hs-CRP.^19^

## OBJECTIVE

The aim of the present study was to identify the amount of physical activity that was required to discriminate normal hs-CRP levels among men and women participating in ELSA-Brasil.

## METHODS

### Study population

In the Brazilian Longitudinal Study of Adult Health (ELSA-Brasil), 15,105 participants of both sexes aged between 35 to 74 years were recruited in six state capitals in Brazil (Rio Grande do Sul, São Paulo, Rio de Janeiro, Belo Horizonte, Vitória and Salvador).^19^^,^^20^ In this analysis, we selected 10,231 participants from the baseline (2008-2010), through exclusion of those who reported that they had previously had cardiovascular disease, were using statins, were diabetic, had not undergone hs-CRP determination, had not given responses to the physical activity questionnaire and presented hs-CRP levels above 10 mg/l, since this level is more suggestive of acute infection.^21^

### Ethical considerations

ELSA-Brasil was approved by the National Commission for Research Ethics and by the research ethics committees of all the six centers involved in these investigations: Universidade Federal da Bahia (under the registration number 027-06); Hospital Universitário da Universidade de São Paulo (669/06); Fundação Oswaldo Cruz (343/06); Universidade Federal de Minas Gerais (186/06); Hospital de Clínicas de Porto Alegre (06-194); and Universidade Federal do Espírito Santo (041/06). All participants signed a free and informed consent form and were guaranteed secrecy and confidentiality of information.

### Data collection

Data were collected by a team of interviewers and measurement technicians who had received training to implement the study protocol. This team was supervised by qualified professionals. Interviews were conducted face-to-face, and anthropometric measurements and blood collection were performed on the same day as the interview.^22^

Sociodemographic variables were determined: sex, educational level (university degree, complete secondary school, completed elementary school or incomplete elementary school), self-identified skin color/race category (white, brown, black, Asian or indigenous), age and functional status (active or retired).

To evaluate nutritional status, the body mass index (BMI) was used, determined as the ratio between weight and height squared. Weight and height were measured by trained personnel in the six centers.

Physical activity was measured using the International Physical Activity Questionnaire (IPAQ), long version, in the domains of physical activity during leisure time (LTPA) and commuting physical activity (CPA). This instrument had previously been validated for use in Brazil. It consists of questions concerning the frequency, duration and intensity of physical activity: moderate-intensity walking within LTPA, i.e. needing some physical effort that makes breathing a little stronger than normal; vigorous-intensity walking within LTPA, i.e. needing great physical effort that makes breathing much stronger than normal; and walking and cycling within CPA.^23^ The amount of physical activity in its different domains was reported in minutes/week and was ascertained by multiplying the weekly rate by the duration of each of the activities. Physical activity was considered to be an activity undertaken for at least 10 minutes/week.

### Laboratory procedures

Blood samples were collected in the six research centers after a mean period of 12 hours of overnight fasting. The samples were stored in dry tubes. To ensure the quality and standardization of results, the processing and analysis of the material were performed in a central ELSA-Brasil laboratory. The hs-CRP levels were measured by means of the quantitative nephelometry method (BN II, Siemens), and the results were expressed as milligrams per liter (mg/l). The hs-CRP measurements were dichotomized regarding cardiovascular risk, in accordance with the recommendations from the Centers for Disease Control and Prevention (CDC) and the American Heart Association (AHA), as high risk (≥ 3 mg/l) and medium/low risk (< 3 mg/l).^24^

### Data analysis

Central trend measurements, frequencies and 95% confidence intervals were calculated for the variables of interest. The predictive power and cutoff points of the LTPA, CPA and combined LTPA + CPA domains for normal levels of hs-CRP were identified from receiver operating characteristic (ROC) curves, which are commonly used to determine cutoff points in diagnostic tests or screening.^25^

Initially, the total area under the ROC curve relating to the amount of physical activity performed (in minutes/week) was identified according to the categories of moderate and vigorous walking in the leisure-time domain (LTPA), walking and cycling in the commuting domain (CPA) and these activities in the summed LTPA + CPA, considering normal levels of hs-CRP. The larger the area under the ROC curve was, the greater the discriminatory power of physical activity for normal levels of hs-CRP also was. The 95% confidence interval was used to determine whether the discriminatory capacity of the physical activity patterns in the domains investigated was or was not due to chance, and whether this should or should not be included in the range of values ≤ 0.50.

To test the differences between the areas under the receiver operating characteristic (ROC) curve, the chi-square test was used, considering a 5% significance level. Sensitivity and specificity were calculated, along with cutoffs for the amounts of physical activity needed to maintain normal levels of CRP, using the Youden index to indicate the optimal cutoff and maximize the overall effectiveness rating for diagnostic purposes.^26^ The data were analyzed using the Stata statistical software, version 12 (Stata Corporation, College Station, United States).

## RESULTS

Out of the 10,231 participants selected for this study, 55.9% were women. The average age was about 50.1 years (standard deviation, SD = 0.1) for men and women together. A higher proportion of the men had educational levels of incomplete and completed elementary school, compared with the women. On the other hand, more than half of the study population had a university degree (55.7%). Regarding employment status, a higher proportion of the women among the employees were retired at the time of the interview (16.6%). It was noteworthy that the frequency of overweight men was higher (44.7%), but more than half of the study population (57.3%) had BMI levels compatible with overweight or obesity. About a quarter of the participants presented high hs-CRP levels (> 3 mg/l) during the investigation period (Table 1).

**Table 1.** Distribution of participants according to selected characteristics and sex. ELSA-Brasil, 2008-2010

Characteristics	Total			Women			Men		
	n	%	95% CI	n	%	95% CI	n	%	95% CI
Age									
Mean (SD)	50.1 (0.1)			50.2 (0.1)			50.0 (0.1)		
35-44	2,866	28.0	(27.1-28.9)	1,552	27.1	(26.0-28.3)	1,314	29.1	(27.8-30.5)
45-54	4,325	42.3	(41.3-43.2)	2,433	42.6	(41.3-43.8)	1,892	41.9	(40.5-43.3)
55-64	2,374	23.2	(22.4-24.0)	1,388	24.3	(23.2-25.4)	986	21.9	(20.6-23.0)
65-74	666	6.5	(6.0-7.0)	344	6.0	(5.4-6.7)	322	7.1	(6.4-7.9)
Educational level									
Incomplete elementary	449	4.4	(4.0-4.8)	166	2.9	(2.5-3.4)	283	6.3	(5.6-7.0)
Completed elementary	580	5.7	(5.2-6.1)	244	4.3	(3.8-4.8)	336	7.4	(6.7-8.2)
Completed high school	3,500	34.2	(33.3-35.1)	1,981	34.6	(33.4-35.9)	1,519	33.7	(32.3-35.0)
University degree	5,702	55.7	(54.8-56.7)	3,326	58.2	(56.9-59.5)	2,376	52.6	(51.2-54.1)
Color/race									
Black	1,487	14.7	(14.0-15.4)	909	16.1	(15.1-17.0)	578	12.9	(12.0-14.0)
Brown	2,900	28.7	(27.8-29.5)	1,524	26.9	(25.8-28.1)	1,376	30.9	(29.5-32.2)
White	5,407	53.4	(52.4-54.4)	3,035	53.6	(52.3-54.9)	2,372	53.2	(51.7-54.7)
Asian	232	2.3	(2.0-2.6)	155	2.7	(2.3-3.2)	77	1.7	(1.4-2.1)
Indigenous	94	0.9	(0.7-1.1)	38	0.7	(0.5-0.9)	56	1.3	(0.9-1.6)
Functional status									
Active	8,811	86.1	(85.4-86.8)	4,769	83.4	(82.4-84.4)	4,042	89.5	(88.6-90.4)
Retired	1,420	13.9	(13.2-14.6)	948	16.6	(15.6-17.6)	472	10.5	(9.6-11.4)
Nutritional status									
Underweight	117	1.1	(0.9-1.4)	64	1.1	(0.8-1.4)	53	1.2	(0.8-1.5)
Normal	4,251	41.6	(40.6-42.5)	2,530	44.3	(43.0-45.6)	1,721	38.1	(36.7-40.0)
Overweight	4,053	39.6	(38.7-40.6)	2,037	35.6	(34.4-36.9)	2,016	44.7	(43.2-46.1)
Obese	1,807	17.7	(16.9-18.4)	1,085	19.0	(18.0-20.0)	722	16.0	(15.0-17.1)
C-reactive protein									
Median (25%-75%)	1.29 (0.66-2.76)			1.42 (0.71-3.15)			1.15 (0.62-2.33)		
< 3 mg/l	7,897	77.2	(76.4-78.0)	4,191	73.3	(72.1-74.4)	3,706	82.1	(80.9-83.2)
≥ 3 mg/l	2,334	22.8	(22.0-23.6)	1,526	26.7	(25.5-27.9)	808	17.9	(16.8-19.0)

ELSA-Brasil = Brazilian Longitudinal Study of Adult Health; SD = standard deviation; CI = confidence interval.

The combined total differs due to loss of information on some variables.

In assessing the amount of physical activity during leisure time that was practiced for at least 10 minutes per week, 42.9% of the study participants of both sexes were found to be inactive. Regarding commuting, it was found that 72.7% were walking and 4.7% were using a bicycle to move for at least 10 minutes per week. Considering the time spent in LTPA and CPA together, 12.6% of respondents reported that they were not doing at least 10 minutes of physical activity during the week (Table 2).

**Table 2.** Distribution of participants according to physical activity for at least 10 minutes/week, domain, intensity and sex. ELSA-Brasil, 2008-2010

Characteristics	Total			Women			Men		
	n	%	95% CI	n	%	95% CI	n	%	95% CI
Leisure-time domain									
Walking									
Yes	3,983	38.9	(38.0-39.9)	2,071	36.2	(35.0-37.5)	1,912	42.4	(40.9-43.8)
No	6,248	61.1	(60.1-61.0)	3,646	63.8	(62.5-65.0)	2,602	57.6	(56.2-59.1)
Moderate									
Yes	2,333	22.8	(22.0-23.6)	1,200	21.0	(19.9-22.1)	1,133	25.1	(23.8-26.4)
No	7,898	77.2	(76.4-78.0)	4,517	79.0	(77.9-80.0)	3,381	74.9	(73.6-76.2)
Vigorous									
Yes	2,504	24.5	(23.6-25.3)	1,055	18.5	(17.4-19.5)	1,449	32.1	(30.7-33.5)
No	7,727	75.5	(74.7-76.3)	4,662	81.5	(80.5-82.5)	3,065	67.9	(66.5-69.3)
Total									
Yes	5,841	57.1	(56.1-58.5)	2,977	52.1	(50.8-53.4)	2,864	63.5	(62.0-64.8)
No	4,390	42.9	(41.9-43.9)	2,740	47.9	(46.3-49.2)	1,650	36.5	(35.1-38.0)
Commuting domain									
Walking									
Yes	7,443	72.7	(71.9-73.6)	4,085	71.4	(70.6-72.6)	3,358	74.4	(73.1-75.7)
No	2,788	27.3	(26.4-28.1)	1,632	28.6	(27.4-29.7)	1,156	25.6	(24.3-26.9)
Cycling									
Yes	478	4.7	(4.3-5.1)	74	1.3	(1.0-1.6)	404	9.0	(8.1-9.8)
No	9,753	95.3	(94.9-95.7)	5,643	98.7	(98.4-99.0)	4,110	91.0	(90.2-91.9)
Total									
Yes	7,542	73.7	(72.8-74.6)	4,105	71.8	(70.6-73.0)	3,437	76.1	(74.9-77.4)
No	2,689	26.3	(25.4-27.1)	1,612	28.2	(27.0-29.9)	1,077	23.9	(22.6-25.1)
Leisure-time + commuting domain									
Yes	8,939	87.4	(86.7-88.0)	4,901	85.7	(84.8-86.6)	4,038	89.5	(88.5-90.3)
No	1,292	12.6	(12.0-13.3)	816	14.3	(13.4-15.2)	476	10.5	(9.7-11.5)

ELSA-Brasil = Brazilian Longitudinal Study of Adult Health; CI = confidence interval.

Areas under the ROC curve that were larger than 0.50 and statistically significant for discriminating normal hs-CRP levels were found in the LTPA domain, independent of intensity, for both sexes. The analysis on physical activity relating to the manner of commuting (walking and cycling) showed that none of the areas under the curve was statistically significant for discriminating normal levels of hs-CRP. However, the sum of physical activity in the two domains showed an area under the ROC curve that was statistically significant for discriminating normal hs-CRP levels in both men and women (Table 3).

**Table 3 .** Areas under the ROC curve and 95% CI of the amount of physical activity as a discriminator for normal hs-CRP levels in adults. ELSA-Brasil, 2008-2010

Domains	Women	Men	Total
PA during leisure time			
Walking	0.53 (0.52-0.55)*	0.53 (0.51-0.55)*	0.54 (0.52-0.55)*
Moderate	0.52 (0.51-0.53)*	0.53 (0.52-0.55)*	0.53 (0.52-0.53)*
Vigorous	0.53 (0.52-0.54)*	0.53 (0.51-0.54)*	0.54 (0.53-0.55)*
Total	0.54 (0.53-0.56)*	0.54 (0.52-0.56)*	0.55 (0.54-0.56)*
PA during commuting			
Walking	0.50 (0.48-0.51)	0.51 (0.49-0.53)	0.50 (0.49-0.52)
Cycling	0.50 (0.50-0.51)	0.50 (0.49-0.51)	0.51 (0.50-0.51)
Total	0.50 (0.49-0.52)	0.51 (0.49-0.52)	0.51 (0.50-0.52)
Sum of PA			
Leisure time + commuting	0.52 (0.51-0.53)*	0.52 (0.51-0.53)*	0.52 (0.51-0.53)*

ROC = receiver operating characteristic; CI = confidence interval; hs-CRP = high-sensitivity C-reactive protein; ELSA-Brasil = Brazilian Longitudinal Study of Adult Health; PA = physical activity.

*Area under the ROC curve showing discriminatory power for normal levels of hs-CRP (CI ≥ 0.50).

**Figure 1.**
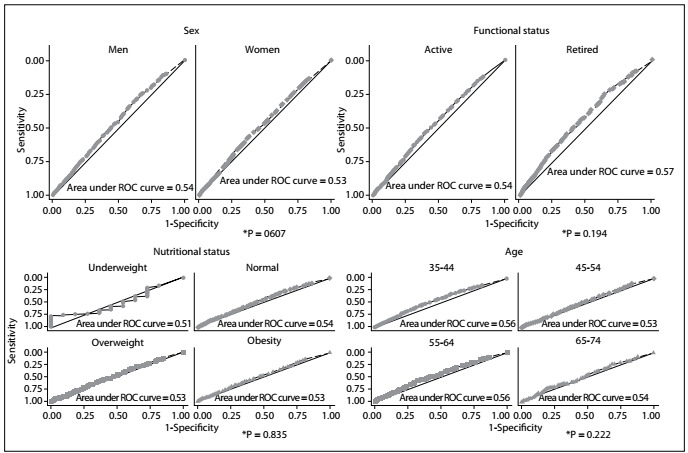
Comparison of the areas under the receiver operating characteristic (ROC) curve of the sum of the amount of physical activity in the two domains studied, for discriminating normal levels of high-sensitivity C-reactive protein (hs-CRP), stratified according to sex, functional status, age and body mass index. Brazilian Longitudinal Study of Adult Health (ELSA-Brasil), 2008-2010.

**Figure 2.**
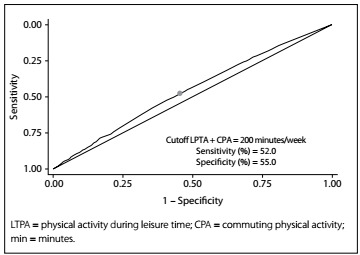
Cutoff point, sensitivity and specificity for the sum of physical activity in the two domains studied (LTPA + CPA), for discriminating normal hs-CRP levels. Brazilian Longitudinal Study of Adult Health (ELSA-Brasil), 2008-2010.

Evaluation according to gender, functional status, BMI and age showed that the areas under the ROC curve relating to the sum of the amount of physical activity in the two domains as a discriminator of normal hs-CRP levels were not statistically significant (Figure 1). The cutoff for the amount of physical activity that best discriminated normal levels of hs-CRP, with sensitivity of 52% and specificity of 55%, was 200 minutes/week for both sexes, when practices during leisure time and commuting were assessed together (Figure 2).

## DISCUSSION

This study demonstrated the discriminatory power of the sum of physical activity in the two domains studied (LTPA + CPA) for normal levels of hs-CRP in adults. It also found that the level of the cutoff for physical activity in minutes per week showed better balance between sensitivity and specificity for discriminating normal levels of hs-CRP in both sexes.

It was observed that a large proportion of this population was not performing at least 10 minutes of moderate or vigorous LTPA, even though the recommended minimum is 150 minutes per week.^27^ The use of bicycles for commuting (4.7%) was lower than in other Brazilian cities and also lower than in European countries such as the Netherlands.^28^^,^^29^ These differences can be explained by various individual, environmental and psychosocial factors, as well as cultural issues.^28^^,^^30^

It was striking that the LTPA domain presented a significant area under the ROC curve (> 0.50) at all intensities (moderate and vigorous physical activity of walking), which translates as discriminatory power for normal levels of hs-CRP. However, other studies with similar methodology, but in which the outcomes were presence of diabetes and visceral fat did not enable recognition of any significant discriminatory power at all intensities of LTPA.^31^^,^^32^ The domain of CPA alone was unable to discriminate normal levels of hs-CRP.

The discussion about the amount of physical activity needed for promotion of good health conditions is not new. In previous studies, results with a dose-response relationship between the level of physical activity and the chance of health problems had also been observed, especially with regard to metabolic and cardiovascular problems.^33^ Studies have shown that less than 150 minutes of physical activity per week is not enough to provide significant benefits regarding protection against some types of health problems.^27^^,^^34^ Nonetheless, contrary to the 2008 recommendations, around 31% of the population of the present study was physically inactive.^35^^,^^36^

The benefits that physical activity can provide in reducing hs-CRP levels have been demonstrated in the literature, but few studies have attempted to identify the discriminatory power of different durations and intensities of physical activity, to keep hs-CRP at normal levels.^16^^,^^17^ In a survey conducted in Finland using a population-based sample, inverse associations between the level of physical activity during leisure time and hs-CRP levels was found for both sexes and between the level of physical activity and hs-CRP levels during commuting for women, even after adjusting for confounding factors.^37^

It was hypothesized in a previous study that one potential route towards reducing hs-CRP concentrations in physically active subjects might be through interleukin levels, especially IL-6 and tumor necrosis factor (TNF-α), but this was not the main subject of that study.^38^ In the present study, it was observed that the amount of physical activity that discriminated normal levels of hs-CRP when evaluated according to functional status, age, obesity and sex did not show any statistically significant difference in area under the ROC curve. It was therefore decided to identify a single cutoff that would cover all the categories evaluated. It was found that only the sum of accumulated physical activity in the two domains studied (LTPA + CPA) had the power to determine the cutoff: 200 minutes per week of physical activity for both sexes. When these domains were analyzed separately, the results regarding the area under the ROC curve were not significant, or the sensitivity and specificity values were not adequate.

Similar results were found in another study conducted in Brazil, in which the outcome studied was diabetes. It was found that 215 minutes of physical activity per week, accumulated over the four domains for women, and 185 minutes of physical activity per week, accumulated over the four domains for men, were the best cutoffs for discriminating the absence of diabetes.^32^

In ELSA-Brasil, it was decided as a methodological strategy only to use the domains of physical activity during leisure time and commuting. However, the International Physical Activity Questionnaire (IPAQ) also includes the domains of physical activity at work and domestic physical activity. The decision not to use these last two domains was made on the basis of studies in the literature, in which a trend of overestimation in the results from these studies in Latin America has been described.^39^

One possible limitation of the present study relates to its use of an indirect measurement for evaluation of physical activity. It may be assumed, for example, that people who are more physically active tend to underestimate the time spent performing their activities, while those who are less active are more prone to overestimation.^40^ It should also be noted that questionnaire-type instruments are more susceptible to recall bias. However, such instruments are used in around two-thirds of countries worldwide, thus enabling benchmarking between them.^35^

It was found in the present study that the sensitivity and specificity values were considered low with regard to diagnostic testing or screening for diseases. However, in considering physical activity as a discriminator, it should not be interpreted as a diagnostic test, but as a form of behavior that presents components and social, biological, psychosocial, cultural and economic factors that make it much more complex. In addition, regular physical activity can bring benefits regarding disease prevention and health promotion.^41^ Similar sensitivity and specificity values were also found in research on physical activity in which the study design was similar but the outcomes were the presence of visceral fat and diabetes. In these studies, the sensitivity ranged from 58% to 68% and the specificity ranged from 52% to 68%.^31^^,^^32^

It can be concluded that accumulated physical activity practices in the leisure time and commuting domains can contribute towards maintaining normal hs-CRP levels. The results suggest that individuals should practice 200 minutes/week of physical activity to maintain hs-CRP at appropriate levels. These results underpin the recommendations of public health policies and programs that aim towards preventing non-communicable diseases, with community interventions, and especially those that stimulate increased physical activity levels in the adult population.
